# Single-cell and genome-wide Mendelian randomization identifies causative genes for gout

**DOI:** 10.1186/s13075-024-03348-z

**Published:** 2024-06-03

**Authors:** Yubiao Yang, Ping Hu, Qinnan Zhang, Boyuan Ma, Jinyu Chen, Bitao Wang, Jun Ma, Derong Liu, Jian Hao, Xianhu Zhou

**Affiliations:** 1 The Second Affiliated Hospital, Guangzhou Medical University, Guangzhou, 510260 China; 2 Department of Orthopedic, Tianjin Medical University General Hospital, 154 Anshan Road, Heping District, Tianjin, 300052 China; 3 Department of Clinical Medicine, Fudan University, Shanghai, China; 4 Medical School Of Ningbo University, Ningbo, China

**Keywords:** Mendelian randomization, Gout, Summary-data-based Mendelian randomization, GWAS, scRNA-seq

## Abstract

**Background:**

Gout is a prevalent manifestation of metabolic osteoarthritis induced by elevated blood uric acid levels. The purpose of this study was to investigate the mechanisms of gene expression regulation in gout disease and elucidate its pathogenesis.

**Methods:**

The study integrated gout genome-wide association study (GWAS) data, single-cell transcriptomics (scRNA-seq), expression quantitative trait loci (eQTL), and methylation quantitative trait loci (mQTL) data for analysis, and utilized two-sample Mendelian randomization study to comprehend the causal relationship between proteins and gout.

**Results:**

We identified 17 association signals for gout at unique genetic loci, including four genes related by protein-protein interaction network (PPI) analysis: TRIM46, THBS3, MTX1, and KRTCAP2. Additionally, we discerned 22 methylation sites in relation to gout. The study also found that genes such as TRIM46, MAP3K11, KRTCAP2, and TM7SF2 could potentially elevate the risk of gout. Through a Mendelian randomization (MR) analysis, we identified three proteins causally associated with gout: ADH1B, BMP1, and HIST1H3A.

**Conclusion:**

According to our findings, gout is linked with the expression and function of particular genes and proteins. These genes and proteins have the potential to function as novel diagnostic and therapeutic targets for gout. These discoveries shed new light on the pathological mechanisms of gout and clear the way for future research on this condition.

**Supplementary Information:**

The online version contains supplementary material available at 10.1186/s13075-024-03348-z.

## Keypoint

This study integrated gout genome-wide association study (GWAS) data, single-cell transcriptomics (scRNA-seq), expression quantitative trait loci (eQTL), and methylation quantitative trait loci (mQTL) data for analysis, and explored the gene expression regulation mechanisms of gout.

This study used two-sample Mendelian randomization method to understand the causal relationship between proteins and gout.

This study identified 17 genetic loci associated with gout, including four genes related by protein-protein interaction network (PPI) analysis: TRIM46, THBS3, MTX1 and KRTCAP2.

This study also identified 22 methylation sites related to gout, and genes that may increase the risk of gout, such as TRIM46, MAP3K11, KRTCAP2 and TM7SF2.

This study determined three proteins causally associated with gout by Mendelian randomization (MR) analysis: ADH1B, BMP1 and HIST1H3A.

**Supplementary Information:**

The online version contains supplementary material available at 10.1186/s13075-024-03348-z.

## Introduction

Gout is a prevalent type of metabolic osteoarthritis induced by elevated blood uric acid levels. Uric acid results from the breakdown of purines, which occurs in numerous substances and cells [1–3]. Hyperuricemia occurs when uric acid production increases due to diet and lifestyle, or when uric acid excretion decreases due to kidney dysfunction [4]. At a certain level of uric acid saturation in the blood, needle-like urate crystals form and deposit in the joints, cartilages, tendons, etc., triggering an immune response and inflammation [5].

A high level of is prevalent in mainland China at a rate of 13.3%, affecting approximately 177 million people; gout is prevalent at a rate of 1.1%, affecting approximately 14.66 million people [6]. In China, the prevalence rate is marginally lower than in Europe and the United States, but it has been rising over the past decade. There are also regional variations; the prevalence is generally higher in economically developed, coastal, and urban regions than in economically less developed, inland, and rural regions. This could be due to living conditions, dietary practices, environmental factors, etc [7].

Some investigations indicate that gout has a genetic component. 10–25% of gout patients’ close relatives have hyperuricemia; if one parent has gout, 40–50% of the child’s children will have gout; if parents have gout, up to 75% of the child’s children will have gout [8, 9]. Genome-wide association studies (GWAS) is the main approach for identifying genetic causes of disease, but the majority of GWAS loci are in noncoding regions, making functional annotation and mechanistic explanations challenging [10]. In recent years, single-cell transcriptomics (scRNA-seq) has become an essential instrument for researching diseases in order to obtain insight into their pathogenesis. Unlike traditional aggregate methodologies, scRNA-seq technology can provide gene expression information from individual cells, thereby overcoming the problem of cellular heterogeneity and providing a more precise and comprehensive perspective for studying diseases [11].

Owing to single-cell transcriptomics, eQTL analysis has emerged as a significant instrument for delving deeper into the mechanisms behind gout. The combination of single-cell transcriptomics and eQTL analysis can shed light on the cell-specificity of gene expression regulation by correlating data from single-cell transcriptomics with genetic variations in individuals and pinpointing locations that regulate gene expression.

**Fig. 1:**
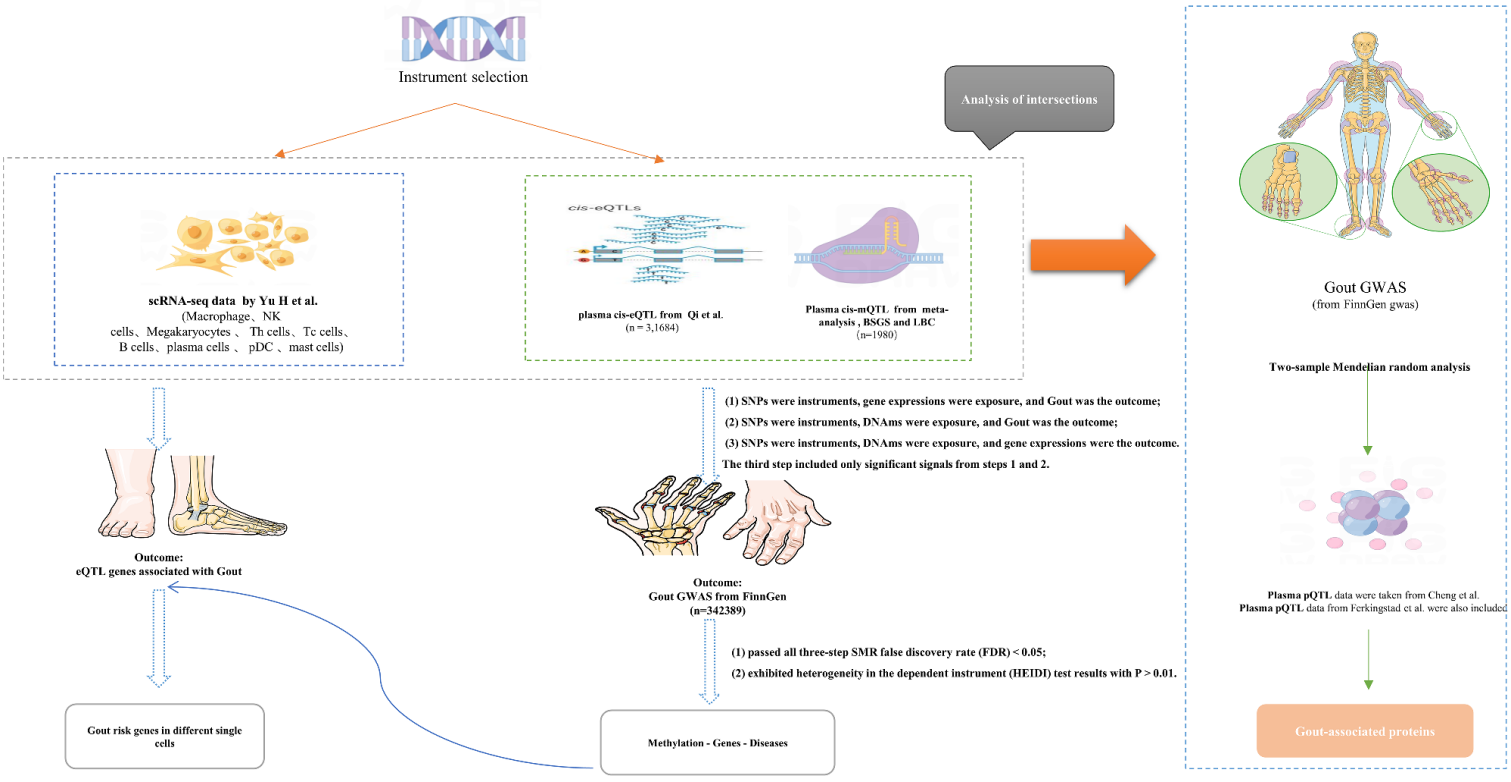
Flowchart of the analyses performed

The present research aims to combine gout GWAS data, scRNA-seq data, and eQTL and mQTL data to investigate the genetic regulation mechanism of gout disease and further our understanding of its pathogenesis. To investigate the dynamic regulatory network in the pathogenesis of gout disease, we will conduct a comprehensive analysis of the expression regulation of gout-related genes in individual cells. And we will comprehend the causal connection between proteins and gout by pQTL and a Mendelian randomization investigation with two samples. Through this study, we expect to elucidate the pathological mechanisms of gout disease and provide new hints and research strategies for gout(Fig. 1).

## Materials and methods

### Gout GWAS data sources

We sourced gout GWAS data from the Finnish database (https://www.finngen.fi/en), encompassing 4607 instances and 335,038 control subjects of European descent. For an in-depth look at the collection of samples, methods of analysis, and findings, kindly refer to the original publication.

### Quantitative trait locus data sources

Using data from the eQTLGen consortium, we retrieved cis-eQTLs located within a 1000 kb range of genetic variants exhibiting a robust correlation with gene expression in relation to blood tissue eQTL data. The eQTLGen consortium includes information on 10,317 SNPs associated with the characterization of 31,684 individuals. eQTLGen does not include variants associated with X and Y chromosome and mitochondrial DNA gene expression levels, however [12].

For mQTL data pertaining to blood tissue, we collected peripheral blood samples from two European cohorts: the BSGS (*n* = 614) and the LBC (*n* = 1366). Using the Illumina HumanMethylation450 microarray, the methylation status of the samples was evaluated. To compile the mQTL summary data, we performed a meta-analysis of BSGS and LBC data. Only DNA methylation probes containing at least one cis-mQTL (*P* < 5 × 10^− 8^) and restricted to SNPs within 2 Mb of each probe were included [13–16].

For pQTL data related to blood tissue, we employed the MR cis-pQTL tool to choose SNPs demonstrating a strong correlation with protein expression from five proteomic databases. We included solely SNPs with a p-value of at least 5 × 10^− 8^ for their association with protein expression. In addition, we integrated the plasma pQTL data of Ferkingstad et al., who conducted measurements of 4907 plasma proteins in a group of 35,559 participants from Iceland [17, 18].

### Sources of single-cell sequencing data for gouty blood peripheral mononuclear cells

We employed the GSE211783 dataset from the GEO database, containing single-cell RNA-sequencing data of peripheral blood from three patients with gout during acute flare and three during remission. Yu H and his team carried out scRNA-seq on PBMC from these patients using 10x Genomics technology, and their results were validated via flow cytometry and LC-MS/MS [19].

### Mendelian randomization analysis based on summary data

We utilized the SMR software application to implement the SMR & HEIDI method, which combines data from GWAS and eQTL studies to assess for multifaceted correlations among levels of gene expression and complex characteristics of interest [20]. For LD calculations, we utilized 1000 Genomes European Reference Data [21]. To gain a deeper understanding of the relationship between eQTLs and mQTLs in terms of disease risk, we conducted a three-step SMR analysis. First, we employed SNPs as instrumental variables, significantly correlated expression of genes as exposure variables, and GWAS as outcome variables. In the second stage, DNAm was used as the exposure variable and GWAS was used as the outcome variable. In the third stage, DNAm was added as the exposure variable, and gene expression was added as the outcome variable. Only significant signals from stages one and two were included in step three [22]. The Benjamini-Hochberg (BH) method was used to compute the FDR of the p-value of the SMR, and FDR SMR 0.05 and heterogeneity HEIDI > 0.01 were used as inclusion criteria for the outcome.

### Two-sample Mendelian randomization analysis

We conducted a two-sample randomization Mendelian analysis utilising “TwoSampleMR” with plasma proteins as the exposure and gout as the outcome. We used the Bonferroni correction to account for multiple testing and a p-value threshold of (*P* < 1.126 × 10 ^− 5^, 0.05/4441) to evaluate the results for telomere length-related proteins.

### Bayesian colocalization analysis

Conventionally, the five postulates of colocalization analysis (Supplementary Methods) are defined as follows: Both the exposure and outcome phenotypes are not associated with the SNP. H1: While the primary phenotype (exposure) correlates with the SNP, the secondary phenotype (outcome) does not. H2: The secondary phenotype (result) correlates with the SNP, whereas the primary phenotype (exposure) does not. Both phenotypes are associated with the SNP, but these associations are distinct. H4: Both phenotypes correlate with the SNP, and the causal SNP is shared by both associations.

Bayesian colocalization analysis utilizing the ‘coloc’ package with default parameters (https://github.com/chr1swallace/coloc) is employed to determine the probability that two characteristics share the same causative variation. As pointed out previously, Bayesian colocalization presents posterior probabilities for the five possibilities regarding whether two characteristics share a single variant. In the present investigation, we calculated the likelihood of the posterior for Hypotheses 3 (PPH3), i.e., both the protein and gout have a relationship with the SNP, but these relationships are independent, and 4 (PPH4), i.e., both the protein and MS have a relationship with the SNP, and these relationships share the same causal SNP. We designated a gene as having colocalization evidence if its gene-based PPH4 was greater than 80%.

### Integrating multi-omics data using summary-data-based Mendelian randomization (SMR) analysis

We utilized the SMR tool (http://cnsgenomics.com/software/smr/) to discover consistent Summary-data-based Mendelian Randomization (SMR) associations across multiple omics. This instrument combines GWAS summary statistics with eQTL or mQTL summary statistics to determine whether particular mutations influence the risk of disease by influencing gene expression or methylation of DNA.

The summary GWAS data for gout were entered as’mygwas. ma’. The methylation quantitative trait loci (mQTL) and expression quantitative trait loci (eQTL) data were respectively entered as’mymqtl’ and’myeqtl’. The SMR instrument processed the input data using the following command:

smr --bfile mydata --gwas-summary mygwas.ma --beqtl-summary mymqtl --beqtl-summary myeqtl --out myplot --plot --probe ENSG00000163462 --probe-wind 500 --gene-list glist-hg19.

The created diagrams provide an overview of the GWAS results displaying the p-values of each SNP, the eQTL results illustrating the effect of each SNP on the expression of genes, and the mQTL results illustrating the effect of each SNP on DNA methylation. This helps to comprehend how variations in gene expression or DNA methylation may affect disease risk.

We intended to identify and validate putative genetic regulatory mechanisms by integrating these results and employing additional statistical analyses (such as the HEIDI test). Such knowledge is essential for comprehending the pathophysiology of diseases and identifying new therapeutic targets.

## Results

### Genome-wide cis-eQTL and SMR analysis of gout outcomes

**Fig. 2:**
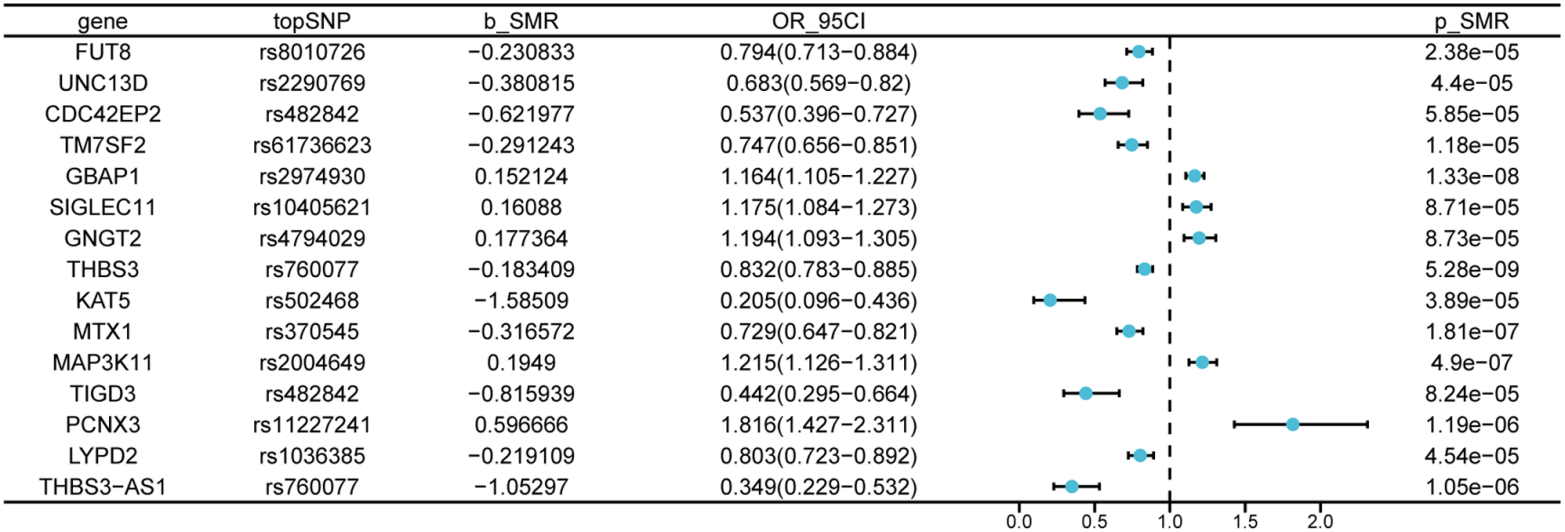
The outcomes of Mendelian randomization, showcasing the relationship between expression quantitative trait loci and the risk of gout, are presented. b_SMR is a marker for the magnitude of effect (β) of the gene variant on the expression of genes. A positive relationship is indicated when β is greater than zero, whereas β less than zero implies a negative relationship. OR, representing odd ratios, is determined from the projection of the causal estimate (β coefficient). The confidence interval, represented by 95%CI, is calculated utilizing β and standard error (SE)

We performed SMR analysis on 15,324 SNPs in the blood that represent pertinent gene expression and gout outcomes in blood. To compensate for the genome-wide kind I errors, we performed FDR correction (*P* < 0.05), which revealed strong evidence of association, followed by HEIDI testing (*P* > 0.01) incorporated in the SMR programme to determine if the associations were caused by sharing causes of variation as opposed to pleiotropy. We discovered 17 gout relationship signatures at distinct genetic loci(Fig. 2, Additional file 1: Table S1). Four genes, TRIM46, THBS3, MTX1, and KRTCAP2, were found to be interconnected via protein-protein interaction network (PPI) analysis(Additional file 2: Figure S1) [23]. A review article, for instance, described the complicated connection between uric acid, gout, and brain disease, and mentioned the THBS3 gene as a gene associated with uric acid metabolism and gout, with variants affecting uric acid excretion and deposition [24]. This is consistent with our finding that a reduction in the standard deviation of THBS3 expression was associated with an 18% risk reduction (beta=-0.18, FDR = 4.12 × 10^− 5^).

### Bayesian co-localization analysis

The SMR analysis has identified 17 genes as gout-causing genes. The posterior probabilities of Hypothesis 4 for THBS3, GBAP1, MTX1, THBS3-AS1, FUT8, UNC13D, LYPD2, and GNGT2 are greater than 0.80, per the Bayesian colocalization analysis (Additional file 3: Figure S2). This suggests that there is a high probability that these genes are associated with gout and that they are likely caused by shared variants. For TM7SF2, CDC42EP2, and TIGD3, however, the posterior probabilities of Hypothesis 3 exceed 0.80. This suggests that these genes are associated with gout, but are most likely controlled by distinct variants.(Table 1).

**Table 1:** Results of eQTL-GWAS co-localization

Exposure	nsnps	PP.H0.abf	PP.H1.abf	PP.H2.abf	PP.H3.abf	PP.H4.abf
THBS3	4641	0	4.60E-06	0	0.011	0.989
GBAP1	4381	0	1.09E-05	0	0.028	0.972
MTX1	4343	2.56E-64	3.66E-05	6.69E-61	0.095	0.905
MAP3K11	4510	0	2.18E-04	0	0.414	0.586
THBS3-AS1	4520	1.56E-18	3.54E-06	4.09E-15	0.008	0.992
PCNX3	4456	4.59E-63	2.21E-04	8.72E-60	0.419	0.581
TRIM46	4623	5.78E-19	1.19E-05	1.51E-15	0.03	0.97
TM7SF2	4651	6.02E-105	4.65E-04	1.14E-101	0.881	0.118
FUT8	7007	0	0.012	0	0.149	0.839
UNC13D	6469	7.85E-130	0.022	2.41E-129	0.066	0.913
KAT5	4419	8.46E-11	1.42E-04	1.61E-07	0.269	0.73
LYPD2	8220	0	0.025	0	0.04	0.935
CDC42EP2	4557	2.49E-60	5.09E-04	4.71E-57	0.966	0.033
KRTCAP2	4623	2.97E-14	2.91E-04	7.77E-11	0.76	0.24
SIGLEC11	6722	0	1.74E-29	0	1	0
GNGT2	5757	0	0.044	0	0.094	0.861
TIGD3	4570	6.50E-35	5.09E-04	1.23E-31	0.966	0.033

The results of the Bayesian colocalization analysis can assist us in evaluating the veracity of these hypotheses, thereby providing crucial hints for future investigation. In particular, the posterior probability represents an estimate of the posterior probability distribution, given the data and prior assumptions. It reflects the likelihood of various hypotheses being supported by the data. Therefore, a greater posterior probability suggests that the hypothesis is more credible given the data.

### Genome-wide cis-mQTL SMR analysis and gene endings

**Fig. 3:**
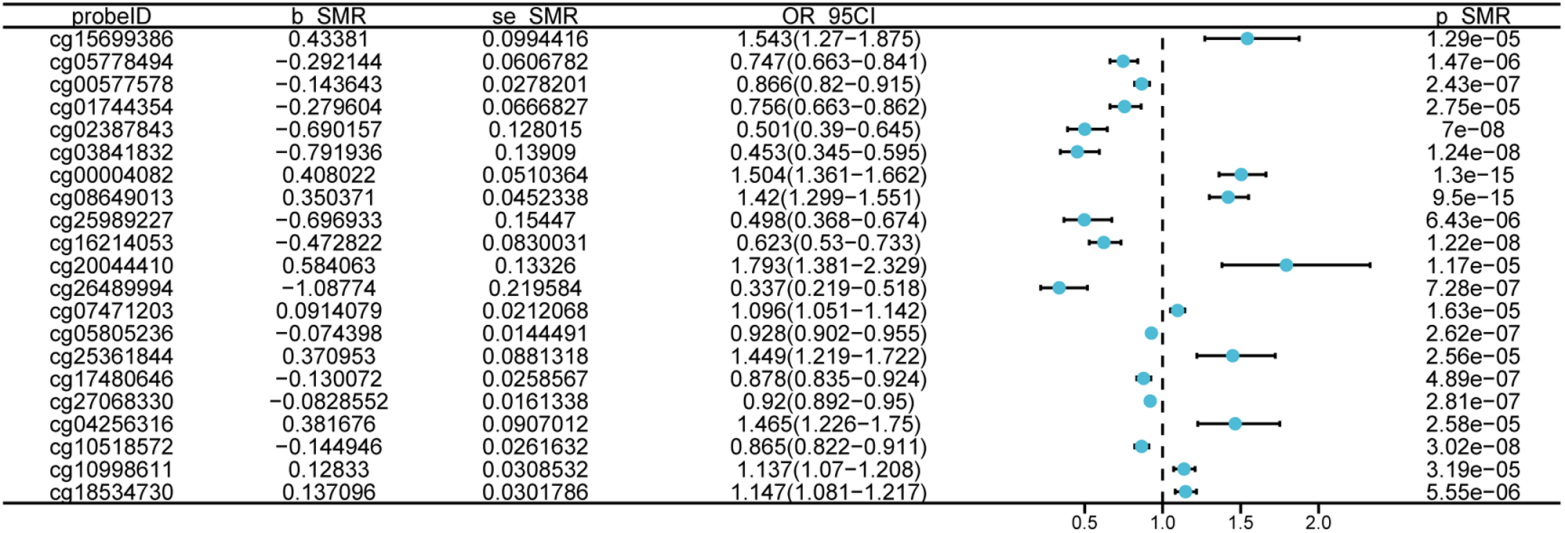
The findings of Mendelian randomization, establishing a connection between methylation quantitative trait loci and the danger of gout, are illustrated. b_SMR symbolizes the impact magnitude (β) of the variant location on DNA methylation. When β is greater than zero, it signifies a positive association, and conversely, β less than zero signifies a negative association. OR, signifying odd ratios, is derived from the forecasted causal estimate (β coefficients). The term 95%CI represents confidence boundaries, ascertained using β and standard error (SE)

To further elucidate the pathogenesis of gout, an SMR analysis, FDR correction, and HEIDI test were conducted between blood mQTL and gout. We identified 22 methylation sites associated with gout, in which multiple methylation sites on SLC2A9 and SIPA1 were regulated, thereby influencing gout disease (Fig. 3, Additional file 4: Table S2). For instance, elevated DNAm at cg25361844 increased disease risk (beta = 0.37), whereas decreased DNAm at cg17480646 increased disease risk (beta = 0.13). Intriguingly, a study of European and Polynesian populations discovered that a prevalent variant in the ABCG2 (rs2231142) was positively associated with hyperuricemia and gout, meaning that populations carrying alleles with this variant had increased uric acid levels and a higher likelihood of gout [25]. However, in the present research, the most significant SNP in this locus, ABCG2, rs10011796, was associated negatively with gout risk. This is an intriguing discovery that warrants further study. In addition, since it is already known that methylation of genes affects gene expression, we mapped gene methyl to expression via sharing variation in genetics and performed an SMR study of the causative connection among methyl and translation of relevant genes.

### Analysis of blood eQTL and mQTL with SMR of gout disease

**Fig. 4:**
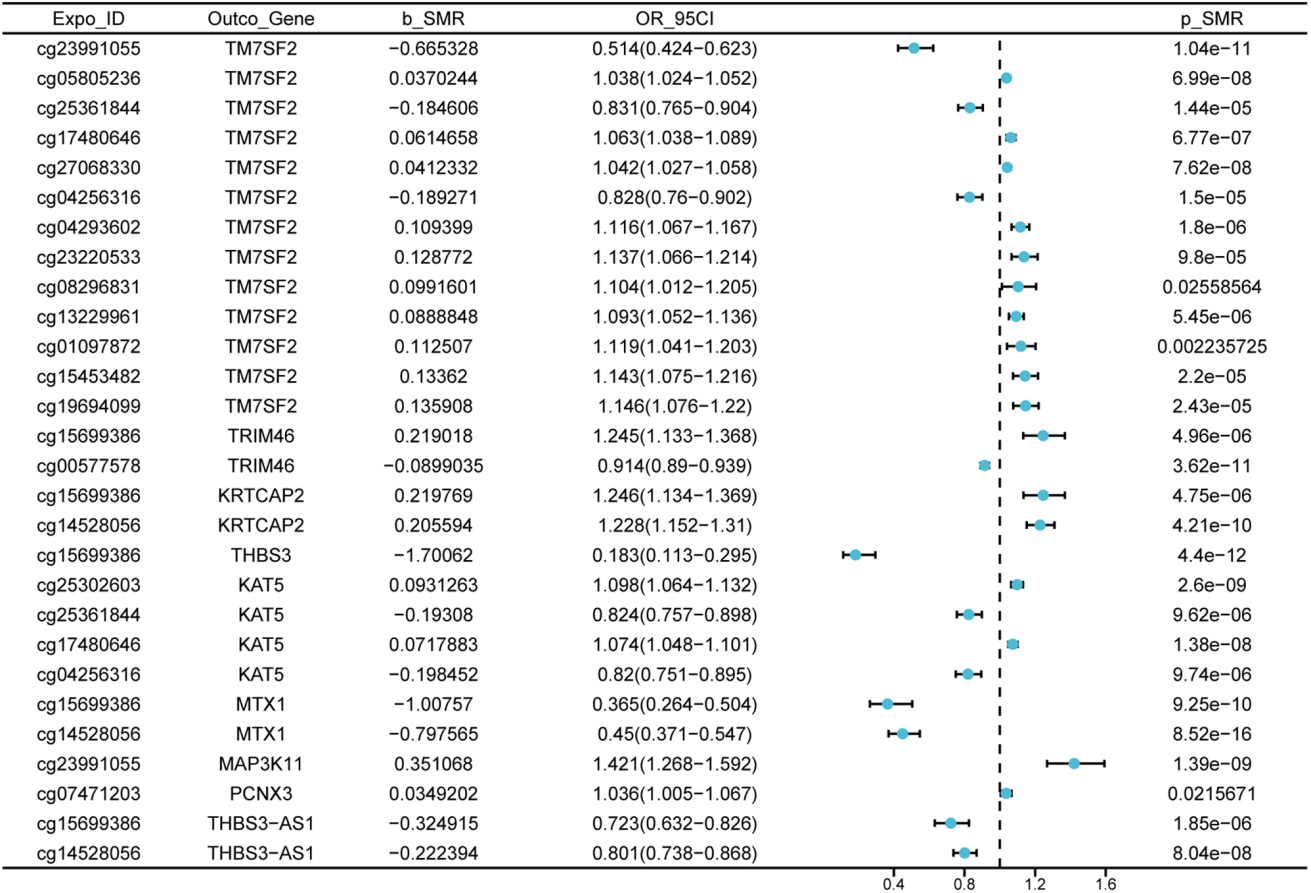
The outcomes from Mendelian randomization, illustrating the correlation between methylation quantitative trait loci and expression quantitative trait loci, are reported. The influence magnitude (β) of a DNA methylation variant location on gene expression is indicated by SMR. A positive association is suggested when β is greater than zero, while a negative association is implied when β is less than zero. OR, the acronym for odd ratios, is computed based on the projected causal estimate (β coefficients). The term 95%CI signifies confidence ranges, which are derived using β and standard error (SE)

In accordance to the three-step SMR study described in the methodology part, we filtered out key disease-related signals. We discovered that TRIM46 had a positive correlation with gout (beta = 1.34); therefore, upregulation of DNAm on the cg15699386 locus would result in an increase in TRIM46 expression (beta = 0.24), which would increase the risk of gout development (beta = 0.43). High DNAm expression at two other loci on the TRIM46 gene (cg05778494, cg00577578) exhibits a negative correlation with this gene’s expression (beta=-0.20, beta=-0.08) (Fig. 4, Additional file 5: Table S3).

Five DNAm sites within the open reading frame (ORF) are substantially linked to TRIM46 and Gout, two of which are in the promoter area region and three in the enhancer region.

**Fig. 5:**
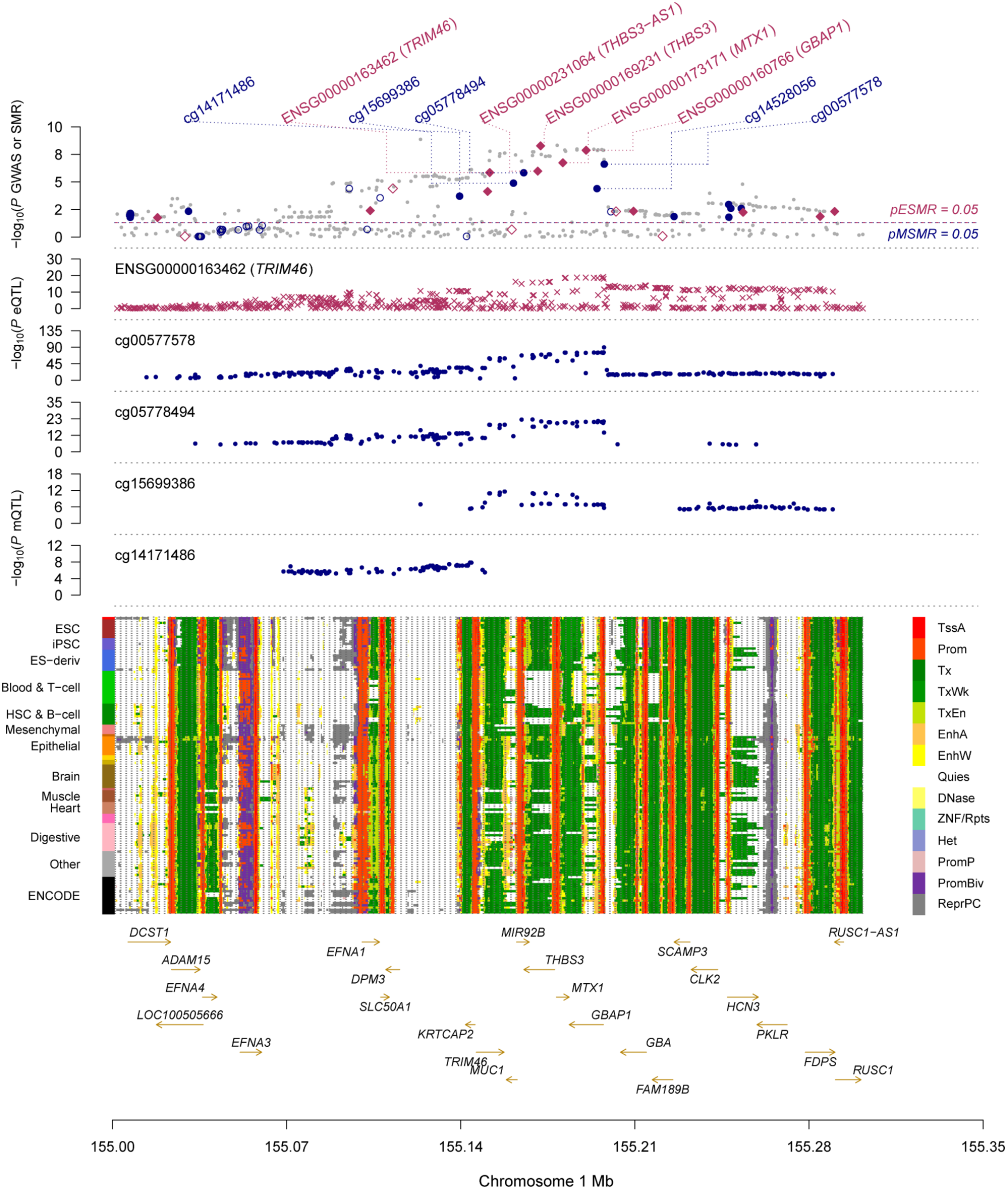
Multifaceted connections between DNA Methylation and gene expression

Using SMR on our omics data, we demonstrate that TRIM46 is a key gene for Gout and may uncover its plausible molecular pathogenesis mechanisms (Fig. 5, Additional file 6: Figure S3).

### Single-cell sequencing analysis

**Fig. 6:**
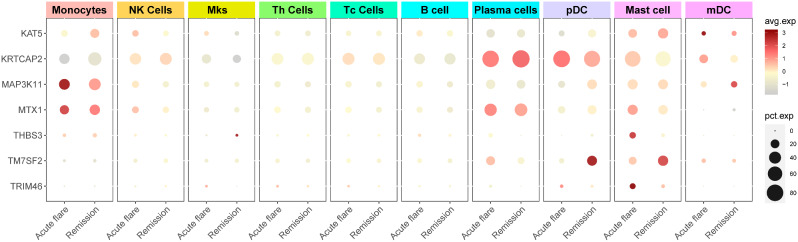
Single-cell sequencing analysis

Eight alleles have been identified that increase the risk of gout, a disease that results from an excess of uric acid in the blood. These genes are predominantly expressed in the monocytes/macrophages, plasma cells, mast cells, and myeloid dendritic cells (MDCs) of the immune system. The genes MAP3K11, KRTCAP2, and PCNX3 influence the function of macrophages, plasma cells, and MDCs, respectively, to increase gout risk. We also discovered a significant correlation between mast cell TM7SF2 expression and gout risk (beta = 1.34) (Fig. 6, Additional file 7: Figure S4).

### Screening the proteome for gout cure-related proteins

We performed a Mendelian randomization analysis to find proteins that are causally linked to gout to find potential therapeutic targets. ADH1B (OR = 0.15, 95% CI = 0.07–0.35), BMP1 (OR = 7.04, 95% CI = 3.35–14.78), and HIST1H3A (OR = 205.85,95%CI = 78.35–540.8) were found to be significantly associated with gout at the Bonferroni-corrected threshold (P 1.719 10 − 6) (Fig. 6, Additional file 8: Table S4). Five SNPs that affect the expression or function of these proteins were correlated with them. However, no protein-protein interaction (PPI) was identified between these three proteins, suggesting that they may act independently in the pathogenesis of gout.

## Discussion

Using GWAS data, scRNA-seq, eQTL, mQTL data, and pQTL with a two-sample Mendelian randomization study, the purpose of this study was to investigate the mechanisms of gene expression regulation in gout disease and improve our understanding of its pathogenesis.

The results demonstrated that mast cells play a crucial role in gout, as they contain the maximum number of disease-associated risk alleles. Prior research has demonstrated a correlation between an increase in mast cells in the synovium of gouty joints and tissue injury [26]. TRIM46 was identified as the most prevalent susceptibility gene with a promotion factor for gout among these mast cells (beta = 1.34). It is noteworthy that a variant site on the TRIM46 gene could potentially affect the activity or expression level of the TRIM46 protein, thereby altering its function in microtubule organization and neuronal polarity maintenance. Such alterations might impair the kidney’s or intestine’s ability to excrete uric acid, leading to increased uric acid levels and a heightened risk of gout [27, 28]. Additionally, TRIM46, by regulating microtubule dynamics, could indirectly affect macrophages’ ability to phagocytose and process monosodium urate crystals (MSUc), which is crucial for modulating the inflammatory response in gout. We have also delved into the role of TRIM46 in the JNK signaling pathway, which has been proven to play a key role in the metabolic and inflammatory response of macrophages triggered by MSUc. TRIM46’s involvement in this pathway could have a comprehensive impact on the production of inflammatory cytokines by macrophages, microtubule dynamics regulation, and cell polarization, thereby regulating the pathological process of gout in terms of uric acid metabolism and inflammatory response [29]. By comparing the unique regulatory role of TRIM46 with existing theories on the pathogenesis of gout, we discovered that TRIM46 might provide new insights into understanding gout inflammation, particularly in analyzing the molecular mechanisms of macrophage responses induced by MSU crystals. This finding not only enriches our understanding of the complex pathological processes of gout but also highlights future research directions, including exploring TRIM46 as a potential target for the treatment of gout and other inflammatory diseases.

In addition, the variant locus on the TRIM46 gene may interact with a history of smoking, thereby increasing the risk of developing gout. By disrupting the equilibrium of uric acid metabolism and inflammatory response, smoking can influence the formation and deposition of urate crystals. Moreover, distinct alleles of the TRIM46 gene may modify the effects of smoking on uric acid levels or inflammatory response, thereby increasing the risk of gout [30]. We speculate that specific variants of the TRIM46 gene might weaken the cell’s ability to resist external oxidative stress, and smoking, as an external source of oxidative stress, could amplify this effect by increasing the level of oxidative stress and disrupting the normal excretion mechanism of uric acid. Additionally, these genetic variants might make cells more sensitive to inflammatory signals in the inflammatory environment caused by smoking, thereby increasing the risk of developing gout. In delving into the interaction between TRIM46 gene variants and smoking history and its role in increasing the risk of gout, we also paid attention to the impacts of other lifestyle and environmental factors, such as dietary habits, alcohol consumption, weight management, and exposure to specific environmental pollutants. These factors could interact with specific variants of the TRIM46 gene to jointly regulate the risk of gout by altering the level of inflammation within the body, the metabolic pathway of uric acid, or the sensitivity of cells to oxidative stress. For instance, unhealthy dietary patterns and high alcohol consumption could exacerbate the overproduction and accumulation of uric acid, while metabolic disorders in overweight or obese conditions could further increase inflammation levels, collectively promoting the development of gout. Simultaneously, long-term exposure to environmental factors like air pollution could increase oxidative stress and inflammation, interacting with TRIM46 variants in regulating the cellular capacity to handle urate crystals, thereby jointly increasing the risk of gout. By integrating the impacts of these lifestyle and environmental factors, we gained a more comprehensive understanding of how TRIM46 gene variants regulate the risk of gout under the combined effects of multiple external factors, emphasizing the importance of considering the complex interactions between genes and the environment in the study of gout pathogenesis. This integrated perspective not only provides new approaches for the prevention and treatment of gout, particularly in terms of lifestyle adjustments and environmental risk management but also directs future research.

In addition, THBS3 and MTX1, both of which are highly expressed in mast cells, share a promoter region. This discovery indicates their potential through interaction to influence the pathogenesis of gout. Specifically, we examined a series of transcription factors, such as STAT3 and NF-κB, which play pivotal roles in regulating inflammation and immune responses and may be directly involved in the expression control of THBS3 and MTX1 [31–33]. These transcription factors might recognize and bind to specific sequences within the shared promoter region of THBS3 and MTX1, thereby modulating their expression. For example, the activation of STAT3 could enhance the expression of THBS3, while the activation of NF-κB could lead to increased expression of MTX1. Such changes in the expression pattern could be crucial for the role of mast cells in the inflammatory process of gout. The interrelationship between these two genes and their combined effect on the development of gout will be investigated in the future.

In addition, we found that the gene KRTCAP2 may function via plasma cells, pDC, and mDC cells to confer gouty disease risk. KRTCAP2 has been linked to autoimmune plasmatic dysplasia, Alzheimer’s disease, and numerous tumor types [34–36]. Additionally, the KRTCAP2 gene may influence the production and clearance of uric acid by modifying the expression as well as the function of the enzyme xanthine oxidoreductase (XOR), thereby impacting uric acid production and clearance [37]. KRTCAP2 may regulate the expression of the xanthine oxidoreductase (XOR) gene by modulating key transcription factors, such as Sp1 or PPARγ [38, 39]. For instance, Sp1 could enhance the activity of the XOR promoter, thereby increasing XOR expression, while changes in KRTCAP2 expression could influence Sp1 activity, thereby indirectly regulating uric acid production. Similarly, by affecting the activity of these transcription factors, KRTCAP2 may not only influence uric acid production but also its clearance in the liver and kidneys. Through this mechanism, the role of KRTCAP2 in the development of gout may be more complex and multidimensional than currently understood.

Moreover, our findings indicate that MAP2K11 has been linked with an elevated risk of gout. Prior study has revealed this MAP3K11 protein is extensively expressed in a variety of tissues, including the nervous system, kidney, liver, pancreas, and lungs [40]. This indicates that the MAP3K11 protein may participate in a variety of physiological processes and signaling pathways. MAP2K11 was identified as a critical component of the p38 signaling path, which has a close connection with uric acid excretion, synoviocyte apoptosis, and autophagy [41, 42]. Inhibition of MAP2K11 expression or activity protects renal function and uric acid excretion by preventing hyperuricemia-induced apoptosis and autophagy in renal tubular epithelial cells [43]. Inhibiting the expression or activity of MAP2K11 in a mouse model study decreased the severity of gouty arthritis by inhibiting the apoptosis and autophagy of synoviocytes and chondrocytes [44]. Involved in the beginning and development of arthritis with gout, MAP2K11 is highlighted as a potential therapeutic target by these findings. Despite the potential efficacy of inhibiting MAP2K11 expression or activity in the treatment of gout, developing such therapies faces a series of challenges and obstacles. First and foremost, identifying safe and effective MAP2K11 inhibitors is a significant challenge, requiring precise control of MAP2K11 activity without affecting other crucial physiological processes. Additionally, evaluating the potential impacts of long-term inhibition of MAP2K11 on other physiological processes, as well as solving drug delivery issues, are challenges that need to be overcome in developing this type of treatment strategy. Future research directions should include further validation of the role of MAP2K11 in gout, especially its specific functions in regulating gout inflammation and renal function. Moreover, the development and testing of small molecule inhibitors targeting MAP2K11, as well as assessing the prospects of these inhibitors in clinical gout treatment, will be important directions for future research.

In addition, a double-sample MR study on protein connections and gout disease revealed associations between three proteins and gout. The strongest correlation was observed with the BMP-1 protein, a metalloproteinase that cleaves numerous matrix proteins, such as collagen, bone morphogenetic proteins, and transforming growth factor (TGF-β) [45]. BMPs as well as TGF- are essential cytokines that control the differentiation and function of numerous immune cells, such as NK cells, T cells, and macrophages [46]. These cells played an important role in the immune system’s reaction to gout as well as can exert proinflammatory or anti-inflammatory effects [47]. BMPs and TGF- also affect uric acid metabolism and excretion, and this, in turn, affects blood levels of uric acid and MSU crystal formation [48]. BMPs, for instance, can inhibit the expression of the uric acid synthesizing enzyme xanthine oxidase (XO) in the liver, thereby reducing uric acid production, whereas TGF- promotes tubular reabsorption of uric acid, resulting in elevated serum uric acid levels [49, 50]. Consequently, the BMP-1 protein emerges as a promising protein target for further research. The BMP-1 protein, through its regulatory effects on uric acid metabolism and immune cell function, offers new opportunities for intervention in the treatment of gout. Compared to current treatments for gout, such as nonsteroidal anti-inflammatory drugs and colchicine, therapeutic strategies targeting BMP-1 could provide more precise treatment options by directly regulating key pathways in uric acid metabolism and inflammatory response, potentially leading to more effective outcomes. Additionally, by modulating BMP-1 activity, the immune system’s response to gout inflammation can be optimized, achieving a balance between pro-inflammatory and anti-inflammatory effects, thus offering comprehensive treatment benefits to patients with gout.However, the development of therapeutic strategies targeting BMP-1 also faces challenges. Identifying BMP-1 inhibitors that are both highly selective and safe, avoiding interference with BMP-1’s role in other physiological processes, presents a significant technical challenge. The long-term efficacy and safety of inhibiting BMP-1 and its signaling pathways remain uncertain, requiring further research to explore the comprehensive role of BMP-1 in the human body. Moreover, ensuring that drugs can effectively reach their target and have good bioavailability is another hurdle that must be overcome in the development process.

Finally, we found by single-cell analysis that MAP3K11, KRTCAP2, PCNX3, and TM7SF2 demonstrate potential significant roles in the pathogenesis of gout. Specifically, MAP3K11, as a serine/threonine kinase, may play a crucial role in the core inflammatory response of gout. Through activating the JNK and p38 MAPK signaling pathways, it not only participates in regulating the activation of immune cells and the production of inflammatory mediators but may also affect gout-specific cellular stress responses, such as the immune response to urate crystals [51, 52]. This process is key to the pathogenesis of gout as it directly involves the initiation and maintenance of the inflammatory response in gout. Although the precise roles of KRTCAP2 and PCNX3 in gout are not fully clear, their biological functions suggest they may indirectly participate in the pathological process of gout through affecting cellular skeletal stability, cell signaling, and stress responses. Specifically, KRTCAP2, as a keratin-associated protein, may play a key role in maintaining the structure and function of immune cells such as macrophages and neutrophils, while PCNX3 may influence the progression of gout inflammation by regulating cellular responses to inflammatory signals [35]. The role of TM7SF2 is more directly associated with the metabolic characteristics of gout. As a factor influencing cholesterol synthesis, TM7SF2 could regulate the levels of cholesterol in cells and tissues, impacting the functionality of inflammatory cells and the production of inflammatory mediators, thereby playing a role in the pathology of gout [53, 54].

To further explore the functions of these genes in the unique inflammatory environment of gout, future experimental designs can focus on the following directions: Firstly, utilizing CRISPR/Cas9 technology to specifically knock out or overexpress these genes in gout models to assess their concrete impact on the inflammatory response of gout. Additionally, in vitro experiments, such as culturing immune cells and exposing them to urate crystals, can be conducted to observe the effects of these genetic modifications on cellular responses. These studies will provide us with an in-depth understanding of the roles these genes play in the pathogenesis of gout, especially how they influence the inflammatory and metabolic processes of gout.

Despite these noteworthy results, the study has some limitations. As only GWAS samples of European provenance were included, the use of existing gout GWAS data may introduce some bias. Consequently, the generalizability of the results to patients with gout of other racial or geographical groups requires further investigation. In our study, we utilized blood-derived samples for single-cell RNA sequencing. While this choice was consistent with other databases we used, ensuring consistency and comparability of data sources, we acknowledge that it may limit the generalizability of our findings, especially considering that other tissues and cell types affected by gout may also be crucial. To address this limitation, we suggest that future research should further broaden the source of samples at the single-cell level, including patients from different ethnicities and geographical backgrounds, and consider using non-blood tissue samples. This approach would not only enhance the universality of our findings but also facilitate our understanding of the specificity of cell types and complexity of intercellular communication in the pathophysiological process of gout. Furthermore, we recommend that future studies adopt multi-time-point sample collection and analysis methods, particularly in single-cell RNA sequencing, to better capture the dynamic changes during the progression of gout. Time-series analysis could provide valuable insights into the evolution of cell states and regulatory networks throughout the development of gout, offering new targets for prevention and treatment. By replicating our single-cell RNA sequencing study across different populations and geographical locations, we can validate the robustness and universality of our findings. This step is crucial for deepening our understanding of the pathogenic mechanisms of gout. In summary, by expanding sample sources, utilizing multi-time-point analysis, and conducting replication studies, we can overcome the limitations of the current research, providing a solid foundation for a deeper understanding of the pathogenic mechanisms of gout and the development of potential therapeutic strategies.

## Conclusion

Our findings suggest that gout is associated with particular genes which are expressed in specific kinds of cells and plays crucial roles within the pathogenesis of the disease. Additionally, we discovered that DNA methylation may modulate the expression and function of these genes, thereby influencing the gout risk. In addition, we identified three proteins that are causally linked to gout and could act as novel targets for the disease’s treatment and detection. These results shed new light on the pathological mechanisms of gout and open up new research avenues. To clarify the relationship between genes, proteins, and gout and their potential therapeutic strategies, additional research is required.

### Electronic supplementary material

Below is the link to the electronic supplementary material.

Supplementary Material 1

Supplementary Material 2

Supplementary Material 3

Supplementary Material 4

Supplementary Material 5

Supplementary Material 6

Supplementary Material 7

Supplementary Material 8

## Data Availability

The datasets used in our study are publicly accessible. Detailed sources can be found in the Methods section of this paper.

## Abbreviations

MR: Mendelian randomization
SMR: Summary-data-based Mendelian Randomization
GWAS: Genome-wide association study
eQTL: Expression Quantitative Trait Loci
mQTL: methylation Quantitative Trait Loci
pQTL: protein Quantitative Trait Loci
SNP: Single nucleotide polymorphism
scRNA-seq: single-cell RNA sequencing
FDR: False Discovery Rate
CI: Confidence interval
OR: Odds ratio
Se: Standard error
IVW: Inverse variance weighting
LD: Linkage disequilibrium
BH: Benjamini-Hochberg
ORF: Open Reading Frame
