# Supplementary figures and images for: Single-cell and genome-wide Mendelian randomization identifies causative genes for gout
Source: Arthritis Res Ther. 2024 Jun 3;26:114. doi: 10.1186/s13075-024-03348-z (PMC11145851; doi:10.1186/s13075-024-03348-z)

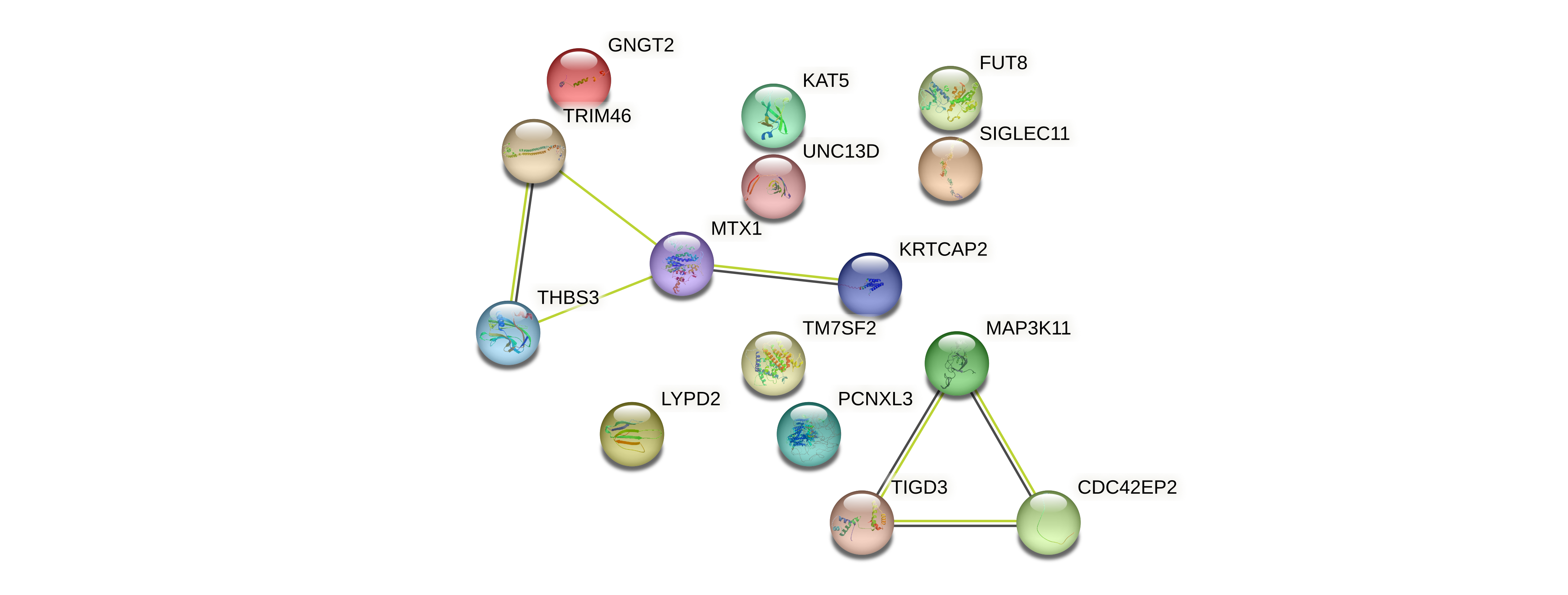

Supplement: Supplementary file 1 — Supplementary Material 1 [file 13075_2024_3348_MOESM1_ESM.png]

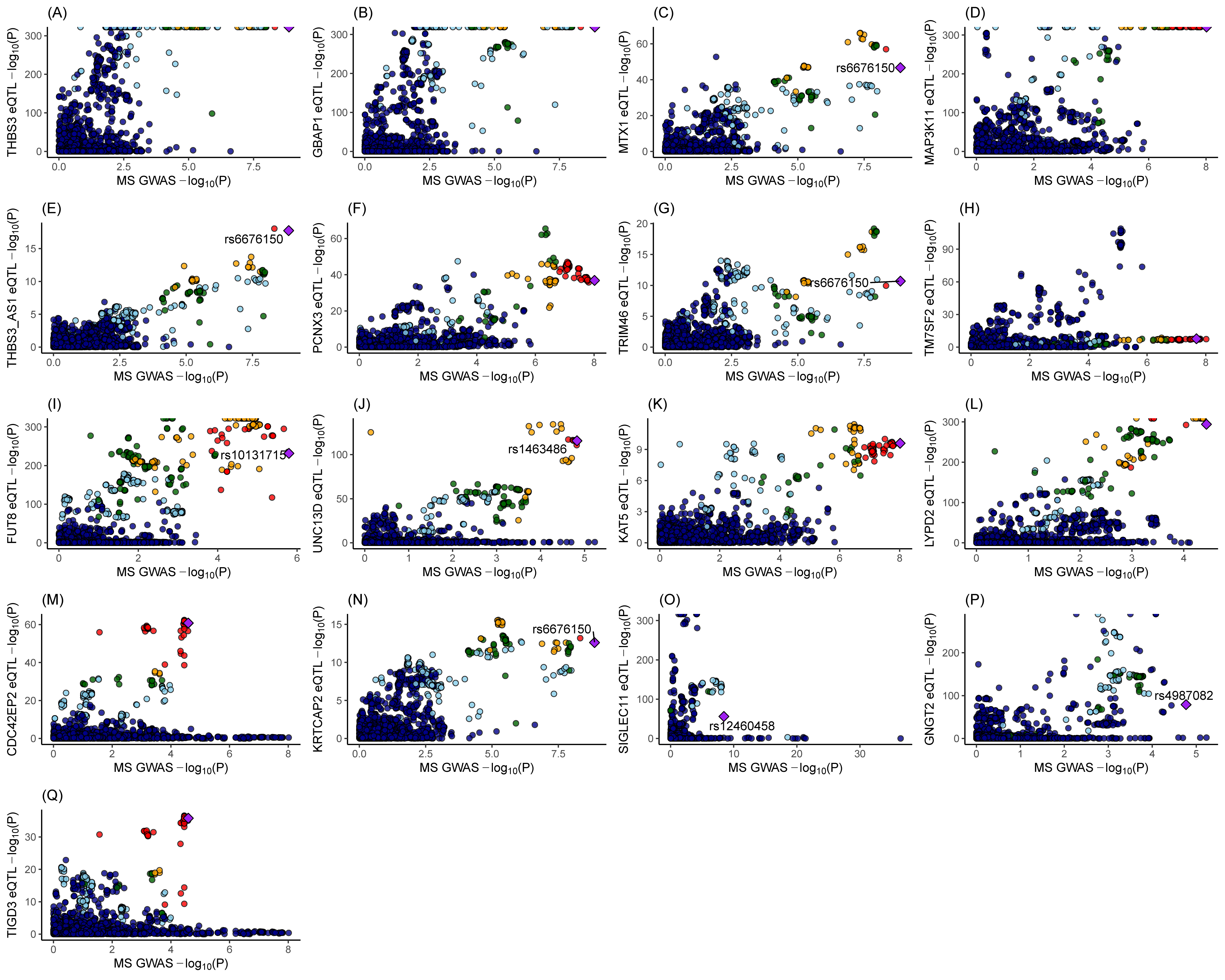

Supplement: Supplementary file 2 — Supplementary Material 2 [file 13075_2024_3348_MOESM2_ESM.png]

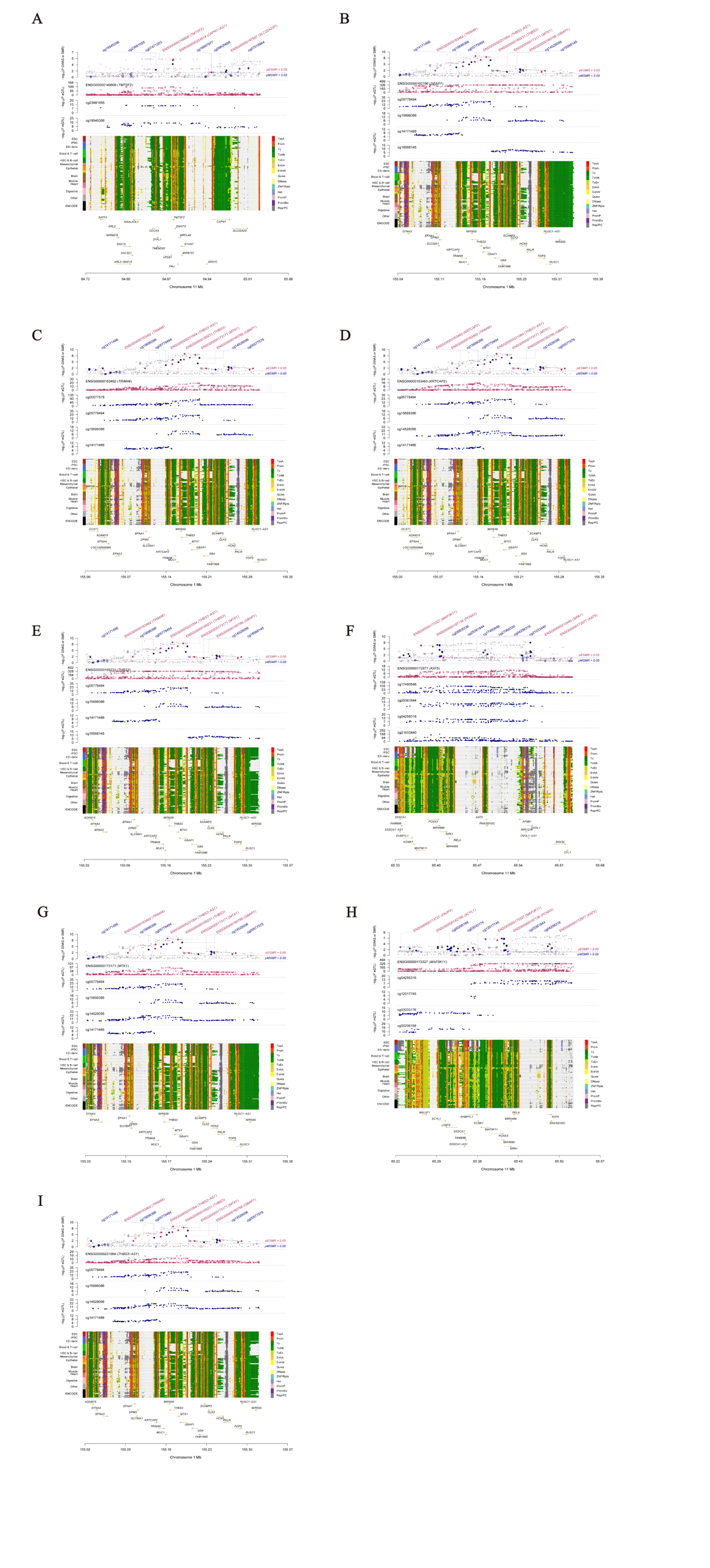

Supplement: Supplementary file 3 — Supplementary Material 3 [file 13075_2024_3348_MOESM3_ESM.png]

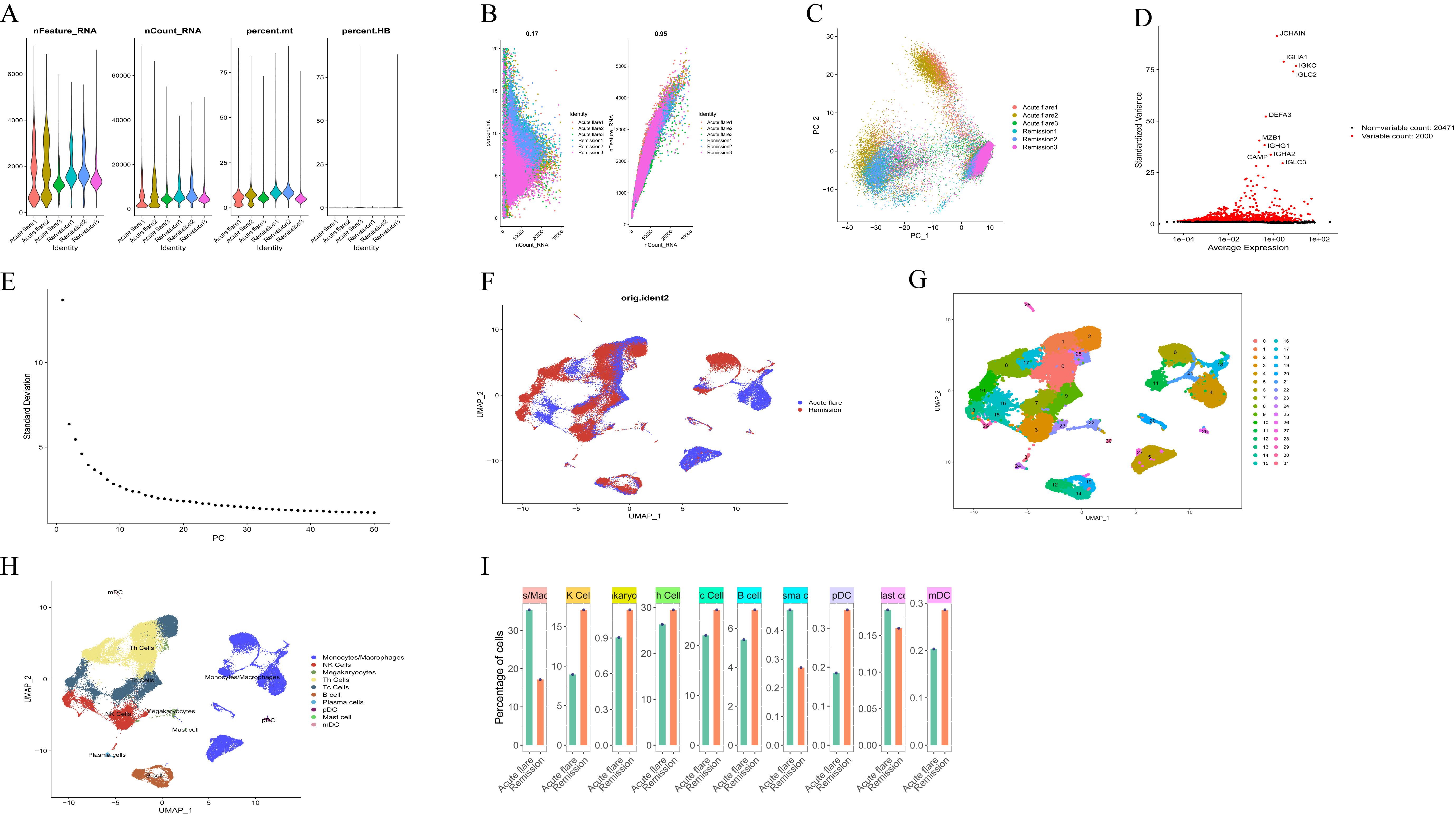

Supplement: Supplementary file 7 — Supplementary Material 7 [file 13075_2024_3348_MOESM7_ESM.png]
